# Pressure ulcer incidence in Dutch and German nursing homes: design of a prospective multicenter cohort study

**DOI:** 10.1186/1472-6955-10-8

**Published:** 2011-04-28

**Authors:** Esther Meesterberends, Ruud JG Halfens, Cornelia Heinze, Christa Lohrmann, Jos MGA Schols

**Affiliations:** 1 Maastricht University, Faculty of Health, Medicine and Life Sciences, Department of Health Care and Nursing Science, School for Public Health and Primary Care (CAPHRI), Maastricht, the Netherlands; 2 Charité Universitätsmedizin Berlin, Department of Nursing Science, Berlin, Germany; 3 Medical University of Graz, Department of Nursing Science, Graz, Austria; 4 Maastricht University, Faculty of Health, Medicine and Life Sciences, Department of General Practice, School for Public Health and Primary Care (CAPHRI), Maastricht, the Netherlands

## Abstract

**Background:**

Pressure ulcers are a common and serious health care problem in all health care settings. Results from annual national pressure ulcer prevalence surveys in the Netherlands and Germany reveal large differences in prevalence rates between both countries over the past ten years, especially in nursing homes. When examining differences in prevalence and incidence rates, it is important to take into account all factors associated with the development of pressure ulcers. Numerous studies have identified patient related factors, as well as nursing related interventions as risk factors for the development of pressure ulcers. Next to these more process oriented factors, also structural factors such as staffing levels and staff quality play a role in the development of pressure ulcers. This study has been designed to investigate the incidence of pressure ulcers in nursing homes in the Netherlands and Germany and to identify patient related factors, nursing related factors and structural factors associated with pressure ulcer development. The present article describes the protocol for this study.

**Methods/design:**

A prospective multicenter study is designed in which a cohort of newly admitted nursing home residents in 10 Dutch and 11 German nursing homes will be followed for a period of 12 weeks. Data will be collected by research assistants using questionnaires on four different levels: resident, staff, ward, and nursing home.

**Discussion:**

The results of the study will provide information on the incidence of pressure ulcers in Dutch and German nursing homes. Furthermore, information will be gathered on the influence of patient related factors, nursing related factors and structural factors on the incidence of pressure ulcers. The present article describes the study design and addresses the study's strengths and weaknesses.

## Background

Pressure ulcers are a common and serious health care problem in all health care settings [1-4]. A pressure ulcer is defined as 'a localized injury to the skin and/or underlying tissue usually over a bony prominence, as a result of pressure, or pressure in combination with shear' [5]. Pressure ulcers can result in a decreased quality of life, an increased need for intensive nursing and medical care and a rise in morbidity and mortality rates [1,2]. Studies around the world have reported large differences in pressure ulcer prevalence rates, varying from 4.7% to 22.9% in hospitals [3,4,6] and 7.7% to 83.6% in nursing homes [6,7].

Results from annual national pressure ulcer prevalence surveys in the Netherlands and Germany, which use the same standardized definitions, instruments and methodology [7,8], have also revealed large differences in prevalence rates between both countries over the past ten years, especially in nursing homes [9,10]. Rates in Dutch nursing homes (30.8%) are reported to be over three times as high compared to those in German nursing homes (8.3%) [10].

When examining differences in prevalence and incidence rates, it is important to take into account all factors associated with the development of pressure ulcers. Various studies have identified a number of patient related factors as risk factors for the development of pressure ulcers. These patient related factors include age [11]; limited mobility and activity levels [12,13]; medical conditions/diseases such as diabetes mellitus [13], Alzheimer disease [14], and cardiovascular diseases [15]; orthopedic problems [13]; medications such as sedatives, analgesics and anesthetics [16]; malnutrition [17]; skin moisture [16]; and urinary and fecal incontinence [18]. Besides patient related factors, nursing related interventions such as the application of repositioning [5] and the performance of nutritional care [19,20] are also linked to the development of pressure ulcers. Furthermore, besides these more process oriented factors, structural factors [21], such as staffing levels, staff quality and the presence and the use of pressure ulcer guidelines [22-24], also play a role in the development of pressure ulcers.

This study has been designed to investigate whether the incidence of pressure ulcers differs between nursing homes in the Netherlands and Germany and, if so, to identify which patient related factors, nursing related factors and structural factors are associated with pressure ulcer development. A detailed description of the methodology of this study is given in this paper.

## Methods/Design

### Study design

This study has been set up as a prospective multicenter cohort study and will be carried out in 10 nursing homes in the Netherlands and 11 nursing homes in Germany. All newly admitted residents in these nursing homes who fulfill the inclusion criteria will be included for participation. In total, 600 residents (300 in each country) will be recruited. Residents who give their informed consent are followed for a period of 12 weeks. This period of 12 weeks was chosen because the national prevalence measurement of care problems in the Netherlands in 2007 showed that 18.3% of nursing home residents developed a pressure ulcer within 12 weeks after admission [25].

### Setting

The participating nursing homes in the Netherlands were selected through the 2008 national prevalence measurement of care problems database [26]. This database contains pressure ulcer prevalence rates from 53% of the Dutch nursing homes. In Germany, nursing homes were selected through the government database for the federal states of Berlin and Brandenburg. Nursing homes could be included in the study if they had a capacity of more than 50 beds and, for practical reasons, were located in the regions Limburg or Brabant (the Netherlands) or Berlin and Brandenburg (Germany). Nursing homes that met these inclusion criteria were entered into SPSS version 17, and subsequently 10 Dutch and 11 German nursing homes were chosen at random by using the 'select cases' option. Rehabilitation and palliative wards were excluded from participation in the study, since the average length of time that residents stay on these wards is short.

### Participants

Only newly admitted nursing home residents are included in this study. Residents are not excluded if they have a pressure ulcer, but are excluded from participation if they have an expected nursing home stay of less than 3 months, if they have been diagnosed with a terminal illness or if their informed consent is received later than three weeks after their admission to the nursing home. All newly admitted residents (and relatives for psycho geriatric residents) who meet the inclusion criteria receive an information package and an informed consent letter during their interview on admission to the nursing home.

### Data collection instruments

Data will be collected using questionnaires on four different levels: resident, ward, nursing home and staff level. For the resident, ward and nursing home levels, most questions are adapted from the Dutch LPZ questionnaires [26]. These questionnaires were developed for the yearly national prevalence measurements of care problems, and are based on information gathered from literature reviews and a Delphi panel of pressure ulcer care experts [4]. The reliability and validity of these questionnaires have been proven in earlier research [7]. The following section describes the questionnaires and instruments used on the four different levels.

#### Resident level

On the resident level, a number of different questionnaires and instruments are used. An overview of the different instruments and time points can be seen in Table 1.

**Table 1:** Overview of instruments and questionnaires on resident level per time point

	Resident questionnaire	Pressure ulcer **questionnaire **^**1**^	Braden Scale	Care Dependency Scale (CDS)	Mini Nutritional **Assessment (MNA) **^**2**^
Week 1	x	x	x		
Week 2	X	x	x		
Week 3	X	x	x		
Week 4	X	x	x	x	x
Week 5	X	x	x		
Week 6	X	x	x		
Week 7	X	x	x		
Week 8	X	x	x	x	x
Week 9	X	x	x		
Week 10	X	x	x		
Week 11	X	x	x		
Week 12	X	x	x	x	x

^1 ^Filled in if the resident has one (or more) pressure ulcer(s)

^2 ^Filled in if the resident has lost more than 5% of body weight during the past month

##### Resident questionnaire

The resident questionnaire contains questions about demographic data (sex, age, gender, weight, length), diseases, reason for nursing home admission, medication use, care dependency, mental status, existence of pressure ulcer(s) (yes/no), repositioning, mobilization, skin care and skin inspection, use of mattresses and cushions, nutrition and prevention of malnutrition, and incontinence care.

Answers are obtained by speaking to the residents directly or, if not possible, by consulting a responsible nurse or nursing assistant and/or the resident documentation. Information about the existence of pressure ulcers is collected by physical examinations conducted by research assistants. The resident questionnaire is filled in weekly for each resident for a period of 12 weeks.

##### Pressure ulcer questionnaire

The pressure ulcer questionnaire contains questions (per pressure ulcer) about the location and the duration of the pressure ulcer and the setting where the pressure ulcer developed (in the current nursing home, in another nursing home, in the hospital, at home or elsewhere). For pressure ulcer grading, the grading system of the European Pressure Ulcer Advisory Panel is used [27]. The length and width of the wound are measured with the perpendicular method using a wound ruler in centimeters. The extension of wound undermining and the depth of the wounds are examined using sterile cotton buds. The wound bed is classified as black (necrotic tissue), yellow (fibrin or slough tissue), red (granulation tissue) or pink (epithelial issue). If 100% of the tissue is pink, this indicates a resurfaced wound. The exudate is classified as none, light, moderate or heavy [28]. Furthermore, the pressure ulcer healing process is monitored using the scores of the Pressure Ulcer Scale for Healing (PUSH Tool 3.0) [29]. The wound edge can be described as smooth, rugged, reddened, pale, inflamed or macerated, since no valid or reliable measuring instruments exist [30]. The wound environment can be described as normal, dry, flaky, humid or inflamed. The odour of the exudate can be qualified as conspicuous or inconspicuous and the quality of the exudate as serous, bloody-serous or purulent. Additionally, information is gained about the treatment of the pressure ulcer (primary and secondary) based on Vasel-Biergans (2006) [31] and whether the resident perceives any pain (0-10 Likert scale).

The PUSH tool was developed by the National Pressure Ulcer Advisory Panel as a quick, reliable tool to monitor the change in pressure ulcer status over time. The tool has been validated by several studies and includes the length and width of the wound in cm^2 ^with a scoring range from 0 to 10, the amount of exudate and the tissue type. The total score ranges from 0 to 17 and helps to indicate improvement or deterioration in pressure ulcer healing [29]. The pressure ulcer questionnaire is filled in weekly if the resident has a pressure ulcer. A separate form is used for each pressure ulcer.

##### Braden scale

The Braden scale is a scale used for determining the risk of pressure ulcer development in patients. The scale, developed in 1984, is one of the most used pressure ulcer risk scales and has a proved validity and reliability [32,33]. The scale consists of six subscales: sensory perception, activity level, mobility, nutritional status, skin's exposure to moisture and friction and shear forces. On each subscale (except friction and shear), scores from 1 to 4 can be given, with 4 representing the highest level. On the friction and shear subscale, scores range from 1 to 3. Total Braden scale scores can range from 6 to 23, with lower total scores indicating a higher risk of developing a pressure ulcer [32]. The Braden scale has been translated into Dutch and German, and several studies have shown the Dutch and German versions of the Braden scale to be valid and reliable [34,35]. The Braden scale is filled in for each resident weekly by the research assistants.

##### Care Dependency Scale

The Care Dependency Scale (CDS) provides a framework for the care dependency status of institutionalized elderly people. It was developed in the Netherlands and has proven validity and reliability [36,37]. The CDS measures 15 concepts of human needs: eating and drinking, incontinence, body posture, mobility, day/night pattern, getting dressed and undressed, body temperature, hygiene, avoidance of danger, communication, contact with others, sense of rules and values, daily activities, recreational activities and learning ability. The care dependency is assessed on a five-point Likert scale. A CDS sum score can be computed by adding the item score of the 15 items, and ranges from 15 to 75. Low scores on the scale items indicate that the patient is completely dependent on care; high scores indicate that the patient is almost independent of care [37,38]. The CDS has been translated into several languages, including German [39]. It has been tested in several studies and proven appropriate for use in nursing home practice and for international comparisons of care dependency [38,39]. The CDS is filled in every four weeks (weeks 4, 8 and 12).

##### Mini Nutritional Assessment

The Mini Nutritional Assessment (MNA) is a validated and reliable instrument, with a high sensitivity and specificity, used to identify elderly residents who are malnourished or who are at risk for malnutrition [40,41]. The MNA is composed of 18 items and involves anthropometric, general, dietary and subjective assessments [42]. The MNA classifies individuals into three levels of nutritional status on the basis of scores that range from 0 to 30. A score of 24 or greater indicates satisfactory nutritional status, a score between 17 and 23.5 indicates a risk of malnutrition, and a score below 17 indicates protein energy malnutrition [41]. The MNA is conducted if a resident has lost more than 5% of body weight during the past month.

#### Staff level

The staff questionnaire was developed to measure the knowledge and practice among nurses and nursing assistants regarding pressure ulcer preventive measures [43]. The questionnaire consists of three different parts. The first part contains questions about the respondents' demographic characteristics, such as age, education and years of working experience in the area of care. The second and third parts of the questionnaire contain questions about the knowledge and practice of nurses and nursing assistants regarding pressure ulcer prevention. These questions are based on the 2002 Dutch national guideline on pressure ulcers [44], which divides pressure ulcer preventive measures into two categories. The first category includes 16 measures that are useful to prevent pressure ulcers for all patients at risk, such as ensuring good hygiene. The second category comprises 13 measures that are not useful to prevent pressure ulcers, such as using gel mattresses and pillows.

In the second part of the questionnaire, the respondents are asked to judge all 29 preventive measures whether they would apply these for patients who are at risk for developing a pressure ulcer. The answering categories for each preventive measure are 'always', 'sometimes' and 'never'. In the third part of the questionnaire, the respondents are asked to judge the usefulness of the preventive measures for patients who are at risk for developing a pressure ulcer. Answering categories for each measure are 'useful', 'sometimes useful', 'not useful' or 'do not know'.

#### Ward level

The ward questionnaire contains questions about the type of ward, the number of residents and rooms on the ward, ward specialization, staffing, presence of pressure ulcer guidelines and whether there is a tissue viability nurse working at the nursing home.

#### Nursing home level

The nursing home questionnaire includes questions about the number of residents in the nursing home, type of specialization and certification of the nursing home, staffing (full time equivalents and qualifications), refresher courses in pressure ulcer care (internal and external), quality control (internal and external) and presence of pressure ulcer guidelines.

### Data collection methodology

Research assistants are responsible for collecting the data among the nursing home residents. In total, three research assistants are responsible for the data collection in the Netherlands and eleven in Germany. All research assistants are nurses or physiotherapists and are educated in the area of pressure ulcer care. All have been trained and instructed to collect the data for this study.

The ward questionnaires are to be filled in by the head of the department. The nursing home questionnaires are to be filled in by the nursing home manager. Both questionnaires are to be completed within a month after the start of the study. Both the ward and nursing home questionnaires are sent back to the principal researchers in both countries by means of stamped addressed envelopes. The staff questionnaires are to be completed by nurses and nursing assistants on the participating wards within the first two months of the study. These questionnaires are collected by the research assistants.

### Sample size calculation

A sample size calculation was performed to determine the number of people who develop a pressure ulcer. To detect a 5% difference in pressure ulcer incidence between both countries (alpha = 0.05; beta = 0.2), with a 15% estimated drop-out rate, 562 residents (280 in each country) need to be recruited.

### Data analysis

Data will be checked for outliers and normality. The analysis includes descriptive frequency distributions for all variables. The level of statistical significance is set at alpha 0.01 (two-tailed). The dependent variable is the development of a pressure ulcer during the 12-week study period for each resident. Bivariate analyses, using cross-tabulations and chi-square tests for nominal data and two sample Wilcoxon tests for continuous data, will be performed to compare each suggested predictor with the dependent variable.

The study will use multilevel models in which 12 repeated measures (the level 1 units) are nested within residents (level 2 units). Multilevel models can accommodate data that are unbalanced due to attrition or missing values. Multivariate logistic regression analyses are used to determine the association between resident, treatment, and facility characteristics and the outcome. All statistical analyses will be performed using Predictive Analytics SoftWare (PASW) version 17 from SPSS.

### Ethical considerations

The medical ethical committees of the Maastricht University Medical Hospital and the Charité - Universitätsmedizin Berlin have granted full ethical approval for this study. The privacy of the participating residents is protected, and all data is coded and processed anonymously.

### Time plan

The nursing homes for this study have already been selected. In the Netherlands, 119 nursing homes met the inclusion criteria. In the first stage, eight of the ten selected nursing homes and, in the second stage, two of the four selected nursing homes were willing to take part in the study. In Germany, 288 nursing homes met the inclusion criteria. In the first stage, six of the ten selected nursing homes were willing to take part in the study; in the second stage one, in the third stage two and in the fourth stage two of the eight selected nursing homes were willing to take part in the study. Resident recruitment began in June 2009 (GER) and August 2009 (NL). The study will be completed in September 2011.

## Discussion

This study has been designed to identify whether there are differences in pressure ulcer incidence rates between nursing homes in the Netherlands and Germany. Additionally, the study will reveal whether these differences can be explained by factors related to patients, nursing care or nursing home structure. To date, many studies concerning pressure ulcer care have a cross-sectional or retrospective design; these designs make it difficult to investigate which factors influence the onset of pressure ulcers because they cannot provide insight into causal relationships. The prospective and longitudinal design of this study will allow us to investigate these factors. Moreover, the longitudinal and prospective design is less vulnerable to measurement error, which is a risk in cross-sectional or retrospective studies [45,46].

To the present authors' knowledge, this study is the first to assess patient related, nursing related and structure related factors in one study. Most previous studies on pressure ulcer care have focused on only one of these factors. The present study makes it possible to gain better insight into all factors related to patients, nursing care and structure that may influence pressure ulcer prevalence and incidence rates, and their interactions. Moreover, many studies on pressure ulcer care obtain data about the existence of pressure ulcers by means of nursing documentation, or analyze data gathered by staff working in the health care institutions. In the present study, all information about the existence of pressure ulcers will be gathered by means of concrete physical examinations carried out by external research assistants to ensure the reliability of the data. Furthermore, the intended sample size and the involvement of multiple nursing homes will improve the reliability of the results.

The internal validity of the study results may be limited due to the exclusion criteria. For example, residents who have an expected nursing home stay of less than 12 weeks will not be included. Additionally, it is possible that participating residents have other clinical characteristics than non-participating residents. For example, non-participating residents may have worse physical conditions than participating residents. Nevertheless, the risk for selection bias will be reduced by the participation of multiple nursing homes in both countries. Although nursing homes were selected from specific regions in both countries, the nursing homes are representative for their country as they differ in size, foundation and specialization. Finally, the drop-out rate of residents during the study due to death, admittance to the hospital or transfer to another nursing home may influence the study results. Drop-outs will be documented thoroughly and included in the data analysis to the point of drop-out.

## Competing interests

The authors declare that they have no competing interests.

## Authors' contributions

EM is responsible for the study design, drafting of the manuscript and critical revision of the manuscript; RJGH is responsible for the study design, critical revision of the manuscript and study supervision, and obtained funding for the study; CH is responsible for the study design, critical revision of the manuscript and study supervision in Germany; CL is responsible for the study design and critical revision of the manuscript; JMGAS is responsible for the study design, critical revision of the manuscript and study supervision. All authors have read and approved the final manuscript.

## Pre-publication history

The pre-publication history for this paper can be accessed here:

http://www.biomedcentral.com/1472-6955/10/8/prepub
